# Synthesis of hydrophobic photoluminescent carbon nanodots by using L-tyrosine and citric acid through a thermal oxidation route

**DOI:** 10.3762/bjnano.5.164

**Published:** 2014-09-11

**Authors:** Venkatesh Gude

**Affiliations:** 1 Department of Chemistry, Assam University-Silchar, Assam, 788011, India

**Keywords:** composite materials, fluorescence quenching, hydrophobic, luminescence, upconversion

## Abstract

Hydrophobic photoluminescent carbon nanodots (CNDs) were fabricated by using citric acid and L-tyrosine precursor molecules through a simple, facile thermal oxidation process in air. These CNDs (less than 4 nm in size) exhibited a characteristic excitation wavelength dependent emission and upconversion emission properties and are insoluble in water, but soluble in organic solvents. FTIR and ^1^H NMR analyses showed a selective participation of L-tyrosine molecule during the carbonization process at 220 °C without a disturbance of its benzylic protons and aromatic phenyl ring bearing hydroxy group. TEM and XRD studies revealed a quasi-spherical morphology and poor-crystalline nature of CNDs. Because the presence of the hydroxy group of L-tyrosine is dominating at the surface, these CNDs are also soluble in water under basic conditions. The effects of base and silver nanoparticles on the luminescence properties of CNDs were studied and a quenching of fluorescence was observed. These tyrosine-passivated CNDs are applicable for both biologically and commercially.

## Introduction

The synthesis of fluorescent functional materials raised significant interest in order to understand biological processes such as DNA sequencing, detection of DNA-hybridization, protein sensing, single molecule detection, energy transfer [1]. A special form of carbon (smaller than 10 nm in size) exhibiting fascinating properties are carbon nanodots (CNDs), which are different in their properties from zero-band gap graphene, diamond, and fullerene. Carbon nanodots (CNDs) exhibit properties such as excitation wavelength dependent multicolor emission [2], upconversion photoluminescence (UCPL) [3], resistance to photobleaching, chemical and photochemical stability, low toxicity [4–5], and bio-compatibility. These associated properties of CNDs are exploited mostly in bio-imaging [6–10], bio-sensor [11–12], and photocatalytic applications [13–14]. CNDs that possess a conjugated conducting network of predominantly sp^2^-hybridized carbon atoms with only a little amount of sp^3^-hybridized carbon atoms, along with a distribution of some atoms such as oxygen and nitrogen are sometimes referred to as heterogeneous graphene quantum dots (GQDs) [15].

Many research groups developed synthetic routes to obtain CNDs without using surface passivating agents, by using natural resources like candle soot [6], orange juice [7], banana juice [16], ground soybeans [17], waste paper [18], and paper ash [19]. The as-prepared CNDs are hydrophilic in nature. There are some synthetic routes by using citric acid and some surface passivating agents like L-lysine [15], ethanolamine [20], betaine [21] to obtain hydrophilic CNDs through thermal oxidation in air. Amino acids like histidine, arginine, threonine, and proline are used as source for producing hydrophilic CNDs in the presence of acid or alkali through microwave pyrolysis [22]. A survey of the literature revealed that the majority of the reports deals with the fabrication of hydrophilic CNDs and their use in cell-imaging [23], sensor [24–27], photocatalytic applications [28–29]. Very few reports about hydrophobic CNDs are known [30–31].

Hydrophobic fluorescent probes (e.g., Nile red) were found to be useful for labeling hydrophobic environments in bacteria [32], but the problem associated with organic dyes is photobleaching. CNDs are promising candidates to replace the hydrophobic fluorescent probes because they are resistant to photobleaching and exhibit strong fluorescence [33]. Excellent fluorescent probes based on CNDs were fabricated for a highly sensitive and selective detection of DNA molecules [24], Cu^2+^ ions [25] and dopamine [26]. The advantages of hydrophobic CNDs are the solubility in organic solvents and the availability in powder form. The synthesis and application of hydrophobic CNDs is less explored. Therefore, the motivation of the present work is to contribute in the development of syntheses of hydrophobic CNDs while using an effective and low-cost approach. Because of the solubility of hydrophobic CNDs in organic solvents they can be used in thin film applications [34], and as a dopant in liquid crystal research [35].

Here I demonstrate a simple, effective, facile, and low cost approach to fabricate hydrophobic CNDs by using commonly available laboratory equipment. The CNDs were prepared by using citric acid and L-tyrosine precursors through a thermal oxidation process in air at two temperatures 220 °C and 300 °C, and they exhibit characteristic emission properties. The two chosen temperatures are below the melting point of L-tyrosine (344 °C) and above the melting point of citric acid (154 °C). During the carbonization process a selective transformation of the L-tyrosine molecule was observed and the benzylic protons as well as the phenyl ring bearing a hydroxy group did not participate in the reaction at 220 °C. This tyrosine-passivated CNDs are insoluble in water under normal conditions, but soluble under basic conditions. The luminescence properties of the CNDs were investigated in organic solvents and in water under basic conditions. The basic aqueous solution of the CNDs was used to prepare a composite material with silver nanoparticles (Ag NPs). The luminescence properties of the composite material solution were investigated and a quenching of emission intensity was observed.

## Results and Discussion

### Synthesis

**Scheme 1:**
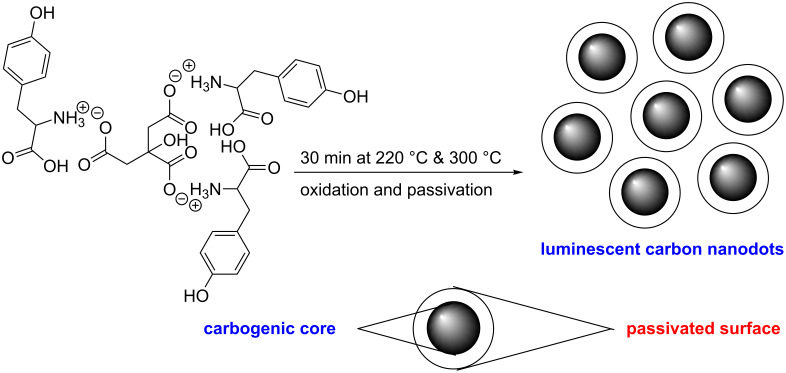
Synthesis of luminescent hydrophobic carbon nanodots.

Hydrophobic photoluminescent CNDs were synthesized by a thermal oxidation process in air using citric acid and L-tyrosine as precursor molecules in a molar ratio of 1:3. Here citric acid serves to make the carbogenic core and tyrosine acts as surface passivating agent. To an aqueous solution of citric acid an acidified (with hydrochloric acid) aqueous solution of L-tyrosine was added slowly in small portions under constant stirring. The resulting solution was evaporated at 100–120 °C to yield a colorless powder. The dried colorless powder was put in a silica crucible and subjected to thermal oxidation in air for 30 min at temperatures of 220 °C and 300 °C (Scheme 1).

During the carbonization process, the evolution of gases and the formation of a dark-brown colored viscous liquid were observed, which indicated that the formation of CNDs is taking place through the formation of amide bonds [20]. The cooling of the reaction mixture after 30 min of reaction resulted in a dark brown solid product, which was dissolved in dry acetone and filtered in order to separate undissolved components such as unreacted reactants and condensation products. The obtained dark brown filtrate was subjected to rotary evaporation in order to produce tyrosine-passivated carbon nanodots (TCNDs) at 220 °C for 30 min, hereafter abbreviated as TCND-1, and at 300 °C for 30 min, abbreviated as TCND-2. At the high temperature the CND yield was clearly lower and a larger amount of undissolved components was found after filtration. The as-prepared TCNDs were subjected to analysis.

### Characterization

**Figure 1:**
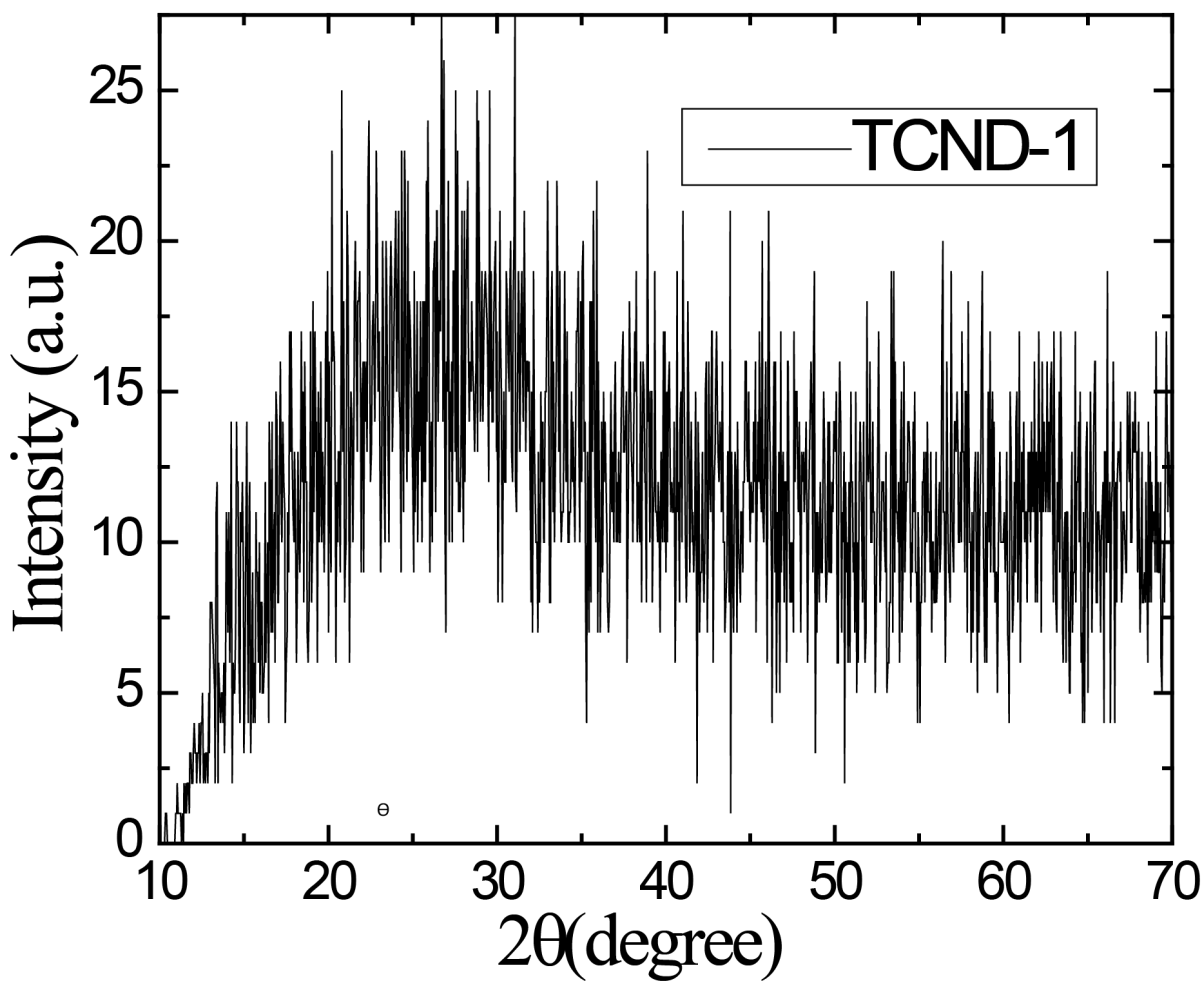
X-ray diffraction pattern of synthesized tyrosine passivated CNDs at 220 °C.

The XRD pattern of TCND-1 exhibits a broad peak centered around almost 24°, which corresponds to a *d*-spacing of about 0.4 nm (Figure 1). This is larger than that of the (002) planes of bulk graphite (0.33 nm). This small difference between synthesized CNDs and bulk graphite is due to the functional groups located at the surface of the layer of the carbogenic core and confirms the amorphous nature of TCND-1.

**Figure 2:**
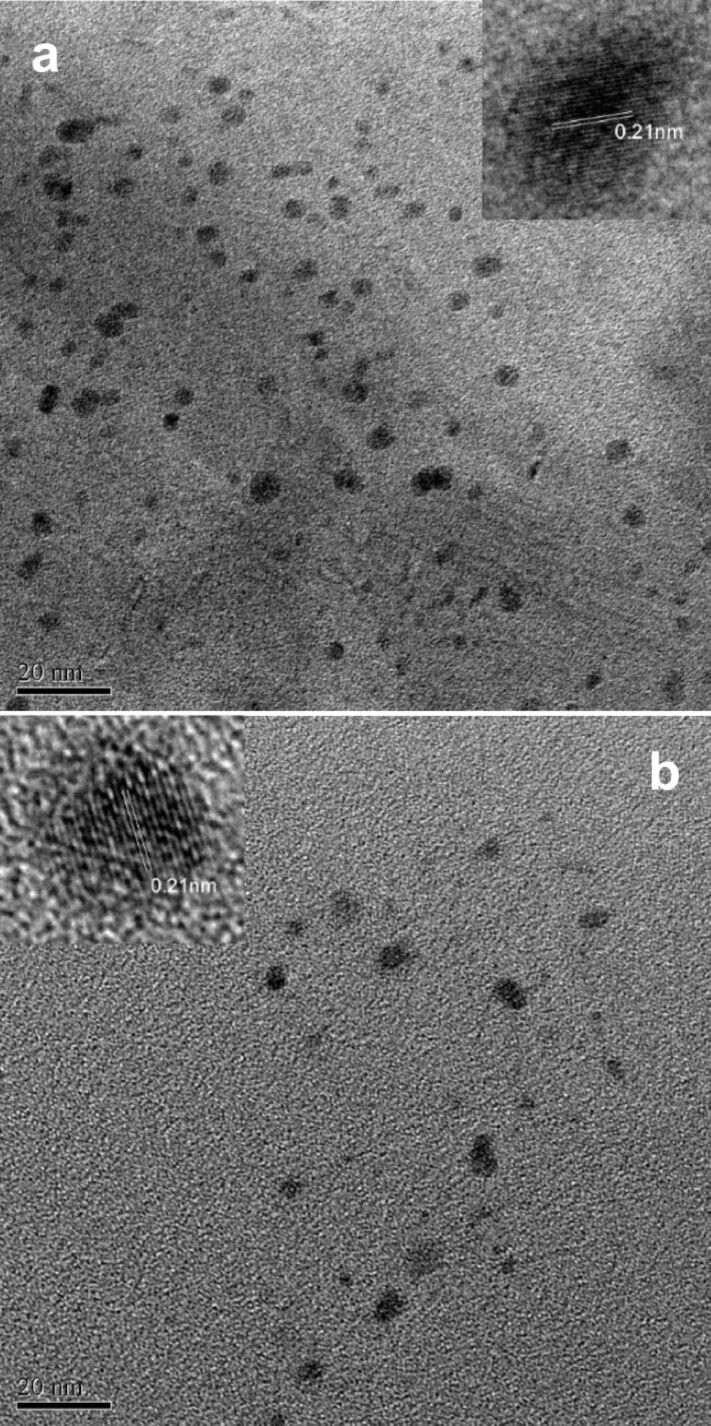
Recorded TEM images of tyrosine-passivated CNDs synthesized at two different temperatures (a) at 220 °C (b) at 300 °C, respectively.

TEM images of the synthesized CNDs revealed the quasi-spherical morphology of both TCND-1 and TCND-2 shown in Figure 2, respectively. In both images the particles appear fully dark colored indicating that the carbogenic core is covered with rigid aromatic rings. The calculated average size of the particles of TCND-1 and TCND-2 are 3.91 and 3.591 nm, respectively. This indicates that at the high temperature more number of bonds are broken during the carbonization process, which leads to the evolution of gas and, hence, a decrease in the size of the CNDs. The lattice-spacing calculated for one of the magnified particles shown in the inset is 0.21 nm, which is closely related to the (100) facet of graphite indicating that the synthesized CNDs are graphitic in nature.

**Figure 3:**
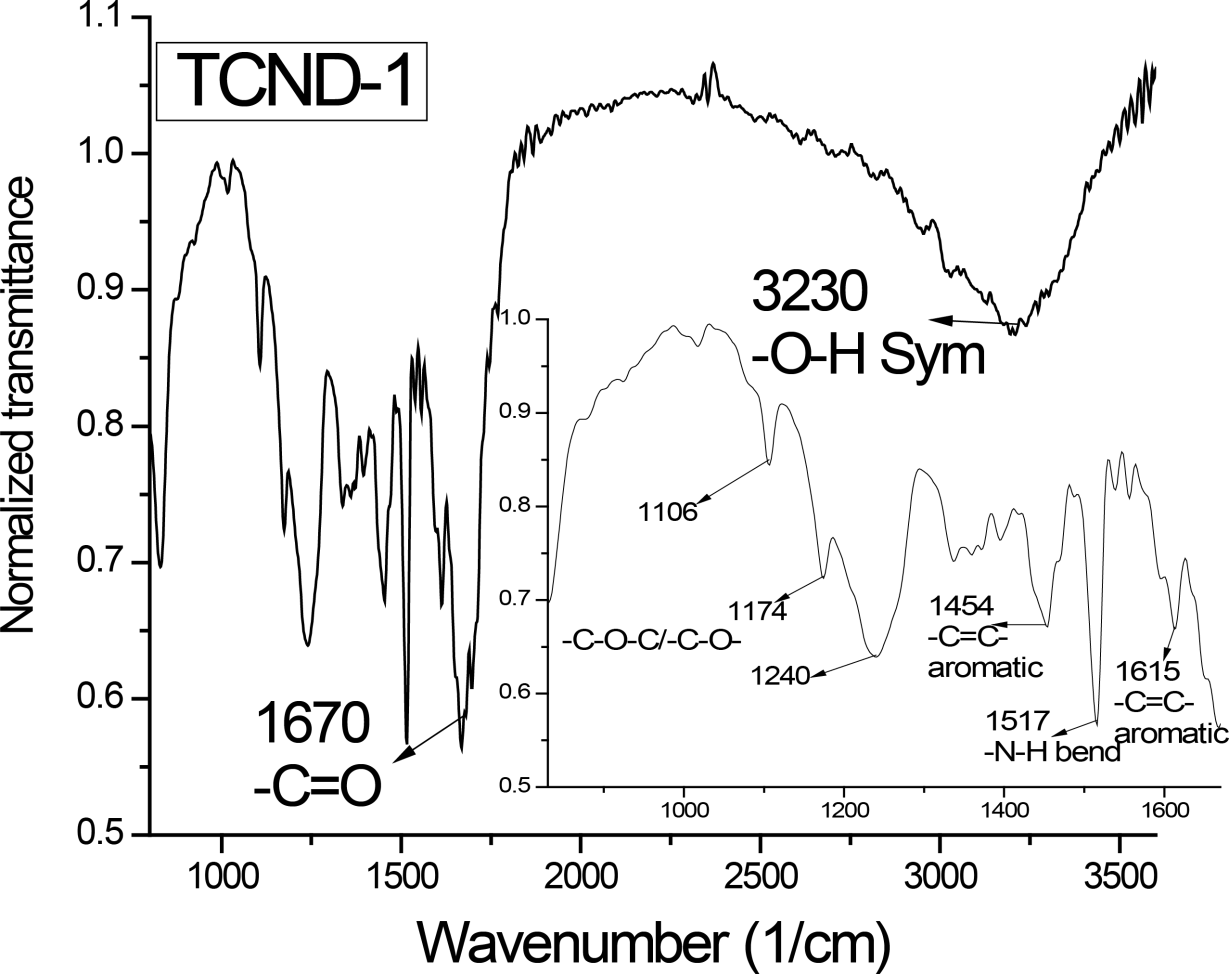
Illustration of recorded FTIR spectrum of TCND-1.

The FTIR analysis of TCND-1 is shown in Figure 3 and confirms the presence of functional groups such as -O–Hν_s_ in the region of about 3400–3150 cm^−1^ and -**CO**NH (-C=Oν_s_ ≈ 1670 cm^−1^) in the CNDs. The signals at 1615 and 1454 cm^−1^ were assigned to the stretching bands of aromatic carbon atoms (-C=C-), the strong signal at about 1517 cm^−1^ was attributed to a combination of a C–N stretching band and a N–H bending band, the weak signals at 1240, 1174 and 1106 cm^−1^ are related to different modes of -C–O–C- and -C–O- groups present in the CNDs [15,20].

**Figure 4:**
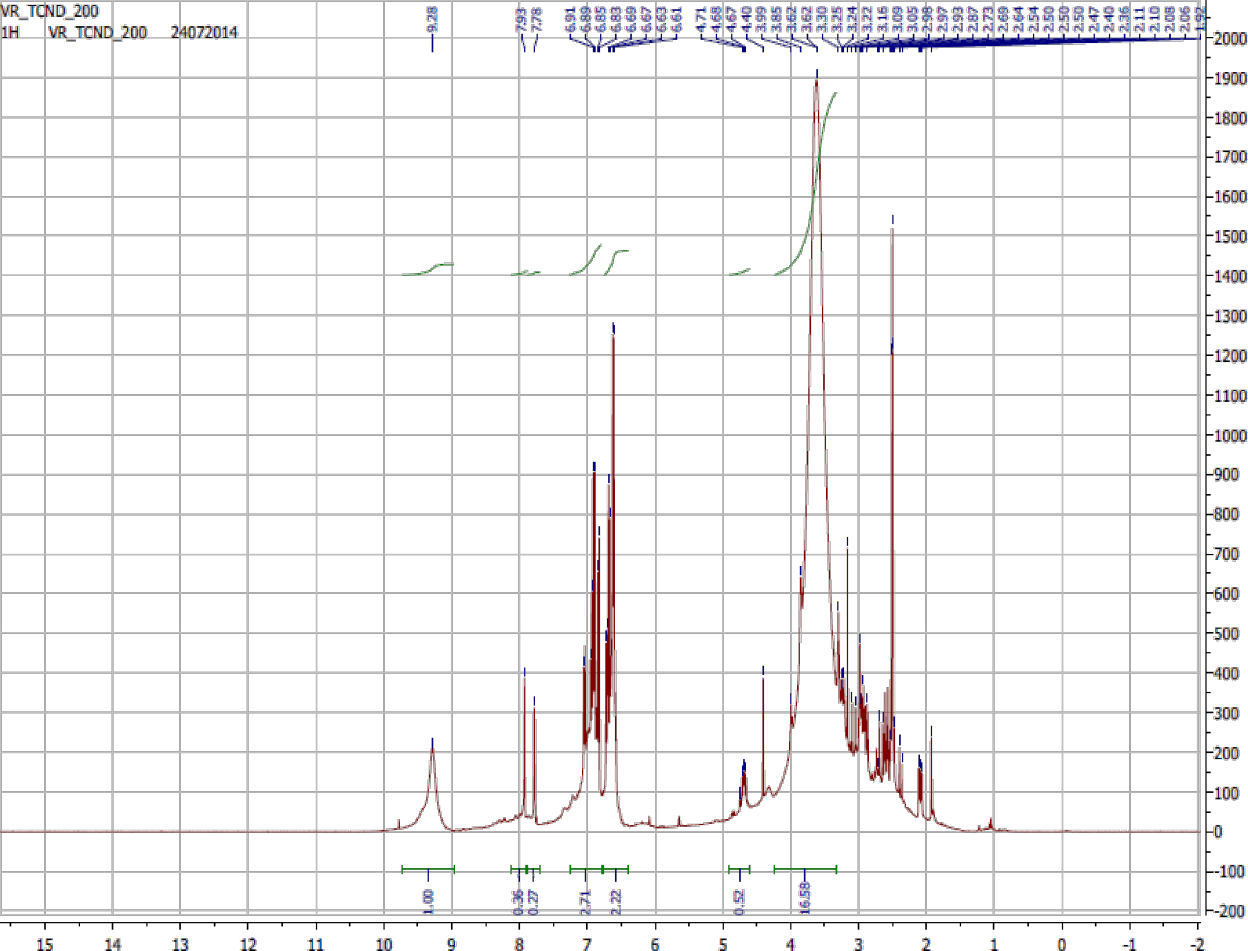
Recorded ^1^H NMR spectrum of TCND-1 in DMSO-*d*_6_.

The ^1^H NMR spectrum of TCND-1 recorded in DMSO-*d*_6_ as solvent is shown in Figure 4. The signals appearing at 2.50 ppm correspond to the solvent and the signals in the range of 2.36–2.98 ppm confirmed the presence of different types of benzylic protons or –C**H**–CONH- or –CONH–C**H**- groups of protons present at the surface of the CNDs. The signals appearing in the range of 3.16–3.85 ppm were assigned to –O–C**H**- groups of protons. The signals continuously appearing in the range of 6.61–6.91 ppm confirmed the presence of aromatic protons. The signals in the range of 7.78–9.28 ppm correspond to phenolic protons. The absence of signals in the region between 10 and 12 ppm indicates that no carboxylic acid (-COO**H**) protons are present in the synthesized CNDs, which evidences the successful separation of CNDs from unreacted citric acid and L-tyrosine.

**Figure 5:**
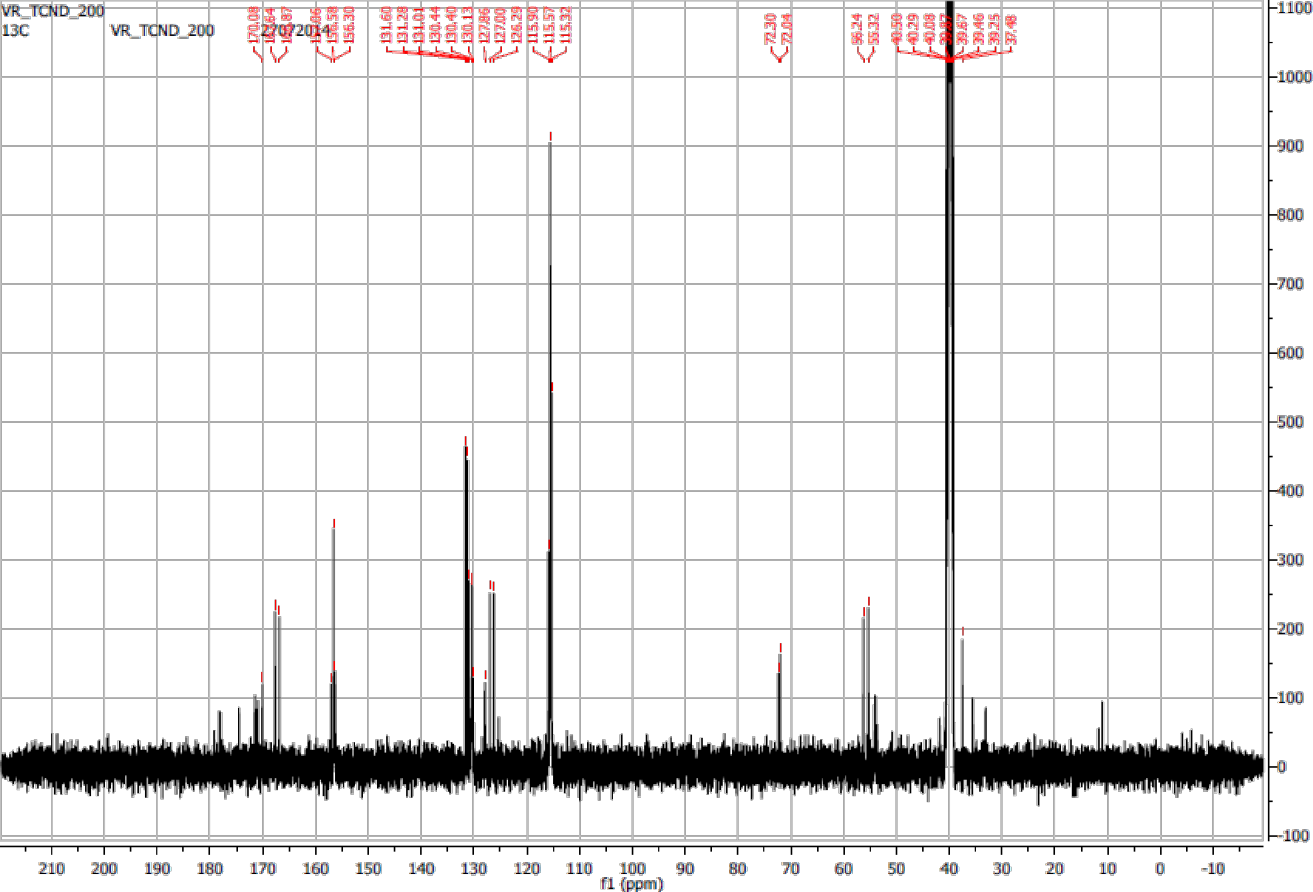
^13^C NMR spectrum of TCND-1 in DMSO-*d*_6_.

The ^13^C NMR spectrum of TCND-1 in DMSO-*d*_6_ is shown in Figure 5. The signal appearing at 39.46 ppm corresponds to carbon present in the solvent molecule. The signals appearing in the region of 40.08–72.30 ppm correspond to carbon atoms that are connected to electronegative atoms such as oxygen and nitrogen. The intense signals present in the region of 115.32–157.06 ppm indicate the presence of sp^2^-hybridized carbon or aromatic carbon atoms connected to hydrogen atoms and the weak signals appearing in the region of 168.87–170.08 ppm evidence the presence of carbonyl carbon atoms. From TEM, FTIR, ^1^H NMR analysis one can understand that the surface of the CNDs is covered by phenol moieties, which indicates a selective participation of L-tyrosine during the carbonization process at 220 °C. The fact that these CNDs are soluble in organic solvents such as acetone, EtOH, MeOH, EtOAc and DMSO but not in highly polar water reflects the hydrophobic nature of the CNDs.

**Figure 6:**
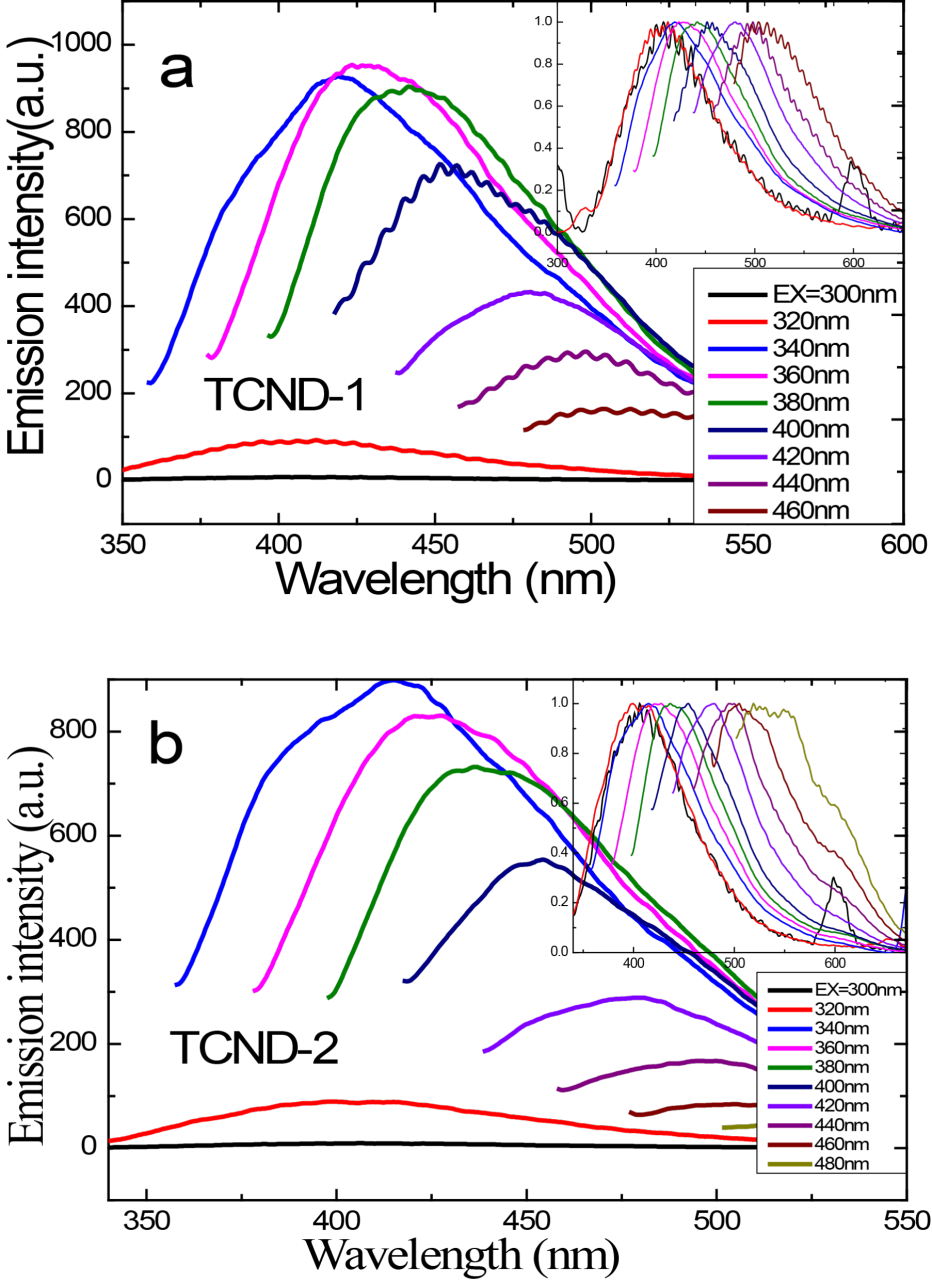
Recorded PL emission spectra of tyrosine-passivated CNDs synthesized at two different temperatures (a) at 220 °C (b) at 300 °C in dry acetone at different excitation wavelengths. The normalized emission spectra are shown in the insets.

**Scheme 2:**
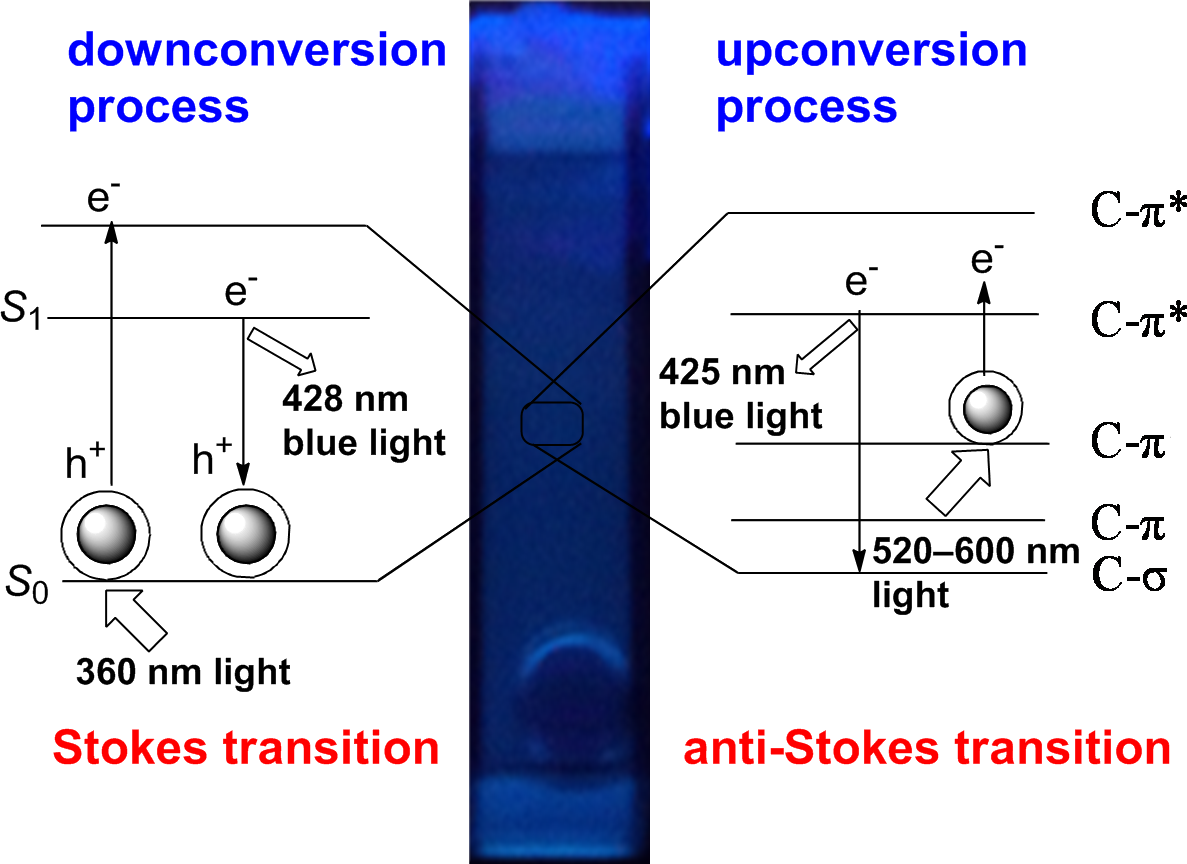
TCND-1 solution exhibited bright blue color emission when exposed to UV light of 360 nm and expected emission mechanism during down and up conversion emission.

The emission spectra of the synthesized fluorescent CNDs recorded in dry acetone (*c* = 2 × 10^−4^ mg/mL) are shown in Figure 6a for TCND-1 and in Figure 6b for TCND-2, respectively. The TCNDs exhibit a characteristic multicolor photoluminescence (PL) emission, which is dependent on the excitation wavelength (λ_ex_). The CNDs solutions exhibited a maximum emission intensity at 428 nm (2.89 eV) for TCND-1 and 413 nm (3.00 eV) for TCND-2, respectively. In both emission spectra excitonic features are readily observed at 360 nm (3.44 eV) for TCND-1 and 340 nm (3.64 eV) for TCND-2, respectively, indicating absorption spectra (Figure S1, Supporting Information File 1) corresponding to blue-light emission (S_1_→S_0_ transition) shown in Scheme 2. Similar of results are observed in both samples in their emission spectra as, under a continuous redshift upon excitation from 340–480 nm, the intensity of the bands decreases the bands become broader in the visible region, respectively. The normalized PL emission spectra shown for both the samples in Figure 6a and b inset indicates the general downconversion emission process and a multicolor emission of CNDs when the excitation wavelength is altered.

The multicolor emission of the CNDs in the visible region that depends on the excitation wavelength appears because different emission sites in the carbogenic core are formed during carbonization process, and the different particle sizes lead to different degrees of conjugation in the core. The exact origin of fluorescence of CNDs is still unclear, but a widely accepted mechanism is the radiative recombination of electron–hole pairs (e^−^–h^+^ shown in Scheme 2) in localized electronic states of the sp^2^ domains, which act as PL centers, dispersed in the sp^3^ matrix. The PL properties of heterogeneous graphene can be tuned on the basis of the ratio of sp^2^ fractions in the sp^3^ matrix, the size, shape of sp^2^ domain and by structural defects [2,5,15,20]. Recently, Cushing et al. reported that the origin of excitation wavelength dependent emission, the continuous red shift and the broadening of bands is due to a “giant red-edge effect” of heterogeneous graphene quantum dots. The giant red-edge effect appears because the relaxation time of the solvent is lower or comparable to the fluorescence life time, which depends on the polarity of the chosen solvent [36]. This explanation contradicts the expected electron–hole pair mechanism of CNDs from earlier reports [2,5]. Therefore, further spectroscopic investigations are required.

The emission quantum yield of the TCND-1 solution was found to be found approximately (3.8%) by using anthracene in ethanol at 340 nm excitation wavelength as reference (Figure S2, Supporting Information File 1). The reason for such a low quantum yield of the TCND-1 solution are the different sizes (inhomogeneity) of the nanoparticles in the solution. This inhomogeneity derives from the carbonization process. Vinci et al. reported that CNDs are often synthesized, modified and studied (in applications) as a mixture of nanoparticles [37]. After high resolution separation of the carbon nanoparticle mixture it was found that the fractionated CNDs exhibited a 5–6 times higher quantum yield than the unfractionated CNDs mixture. The reported results by Vinci et al. suggested that some components (absorbing species) present in the mixture did not exhibit photoluminescence and suppressed the quantum yield of the mixture of CNDs [37].

**Figure 7:**
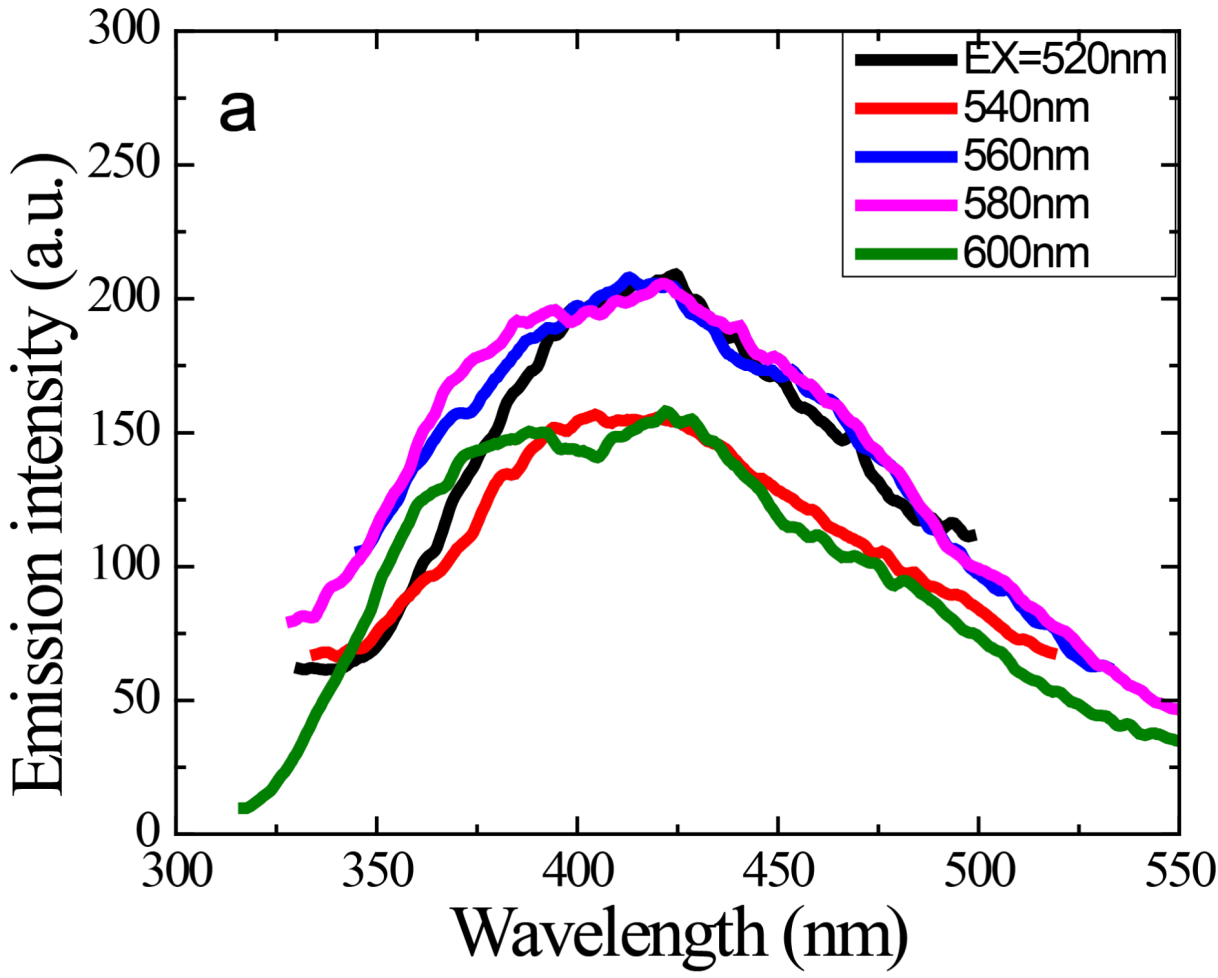
Upconverted PL spectra of TCND-1 in dry acetone at excitation wavelengths in range between 520 and 600 nm.

Another interesting optical property of these tyrosine-passivated CNDs is upconversion photoluminescence (UCPL) when irradiated with wavelengths above 500 nm, which is very important for applications as photocatalyst and for light harvesting applications [2,5]. The recorded PL emission spectra of a freshly prepared TCND-1 solution are shown in Figure 7. Upon excitation by long-wavelength light in the range of 520–600 nm, an upconverted emission peak is observed in the range of 350–500 nm with a maximum emission intensity observed at 425 nm. The energy difference, δ*E*, between the upconverted emission and the excitation light is about 0.5–0.8 eV, which is in good agreement with the δ*E* value below 1.5 eV determined by Hoffmann [2]. It is known that CNDs do exhibit UCPL and I expect that the origin of the upconverted emission peak is the excitation of π-electrons to non-bonding molecular orbitals (π*) with a subsequent direct relaxation to lower energy π-states of the molecule or σ-states of the carbogenic core, followed by an excitation of an electron for normal fluorescence [2]. These transitions (anti-Stokes transition) are shown in Scheme 2. Recently, Wen et al. reported that the origin of upconversion fluorescence is normal fluorescence excited by leaking light from the second diffraction in the monochromator of the fluorescence spectrophotometer [38]. However, the exact origin of UCPL emission of CNDs is unclear and requires more spectroscopic investigations.

**Figure 8:**
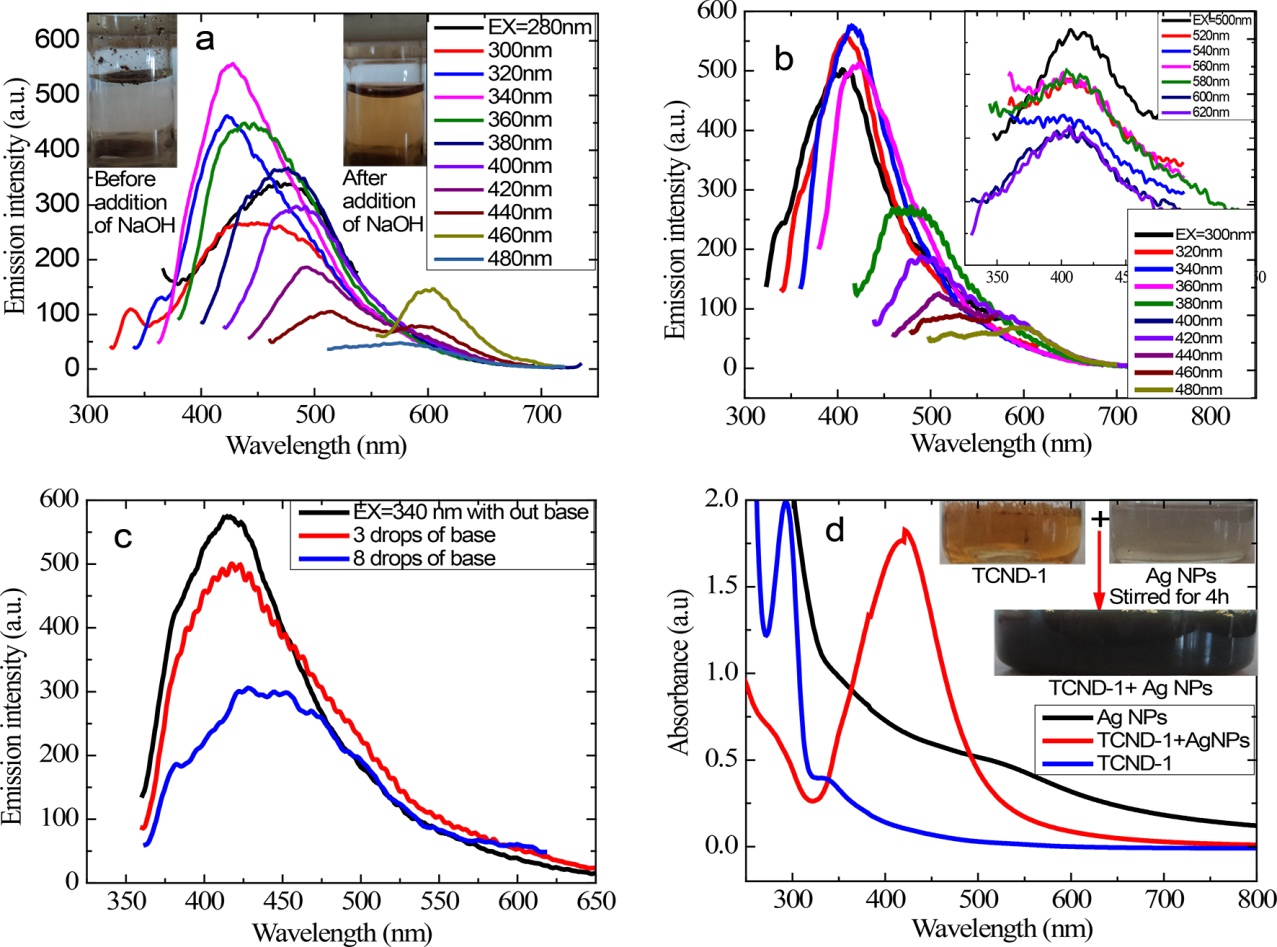
PL emission spectra of TCND-1 at different excitation wavelength (a) in aqueous solution under basic conditions (b) in ethanol solution and upconversion emission spectra shown in inset. (c) Emission spectra of TCND-1 at an excitation wavelength of 340 nm before and after addition of three and eight drops of base in ethanol. (d) Absorption spectra of Ag NPs, TCND-1, and the composite material in aqueous solution under basic conditions, respectively.

TCND-1 is not soluble in water but is soluble in water after the addition of a base. This indicates the formation of CNDs oxide salts and confirms the presence of the hydroxy group of L-tyrosine around the carbogenic core. The recorded PL emission spectra of TCND-1 in aqueous solution are shown in Figure 8a. The maximum emission intensity is observed at an excitation wavelength of 340 nm, which is similar to Figure 6a and all other features described above for TCND-1 are reflected here. This indicates that the addition of 0.4 M NaOH does not influence the emission sites of the carbogenic core but served only to solubilize CNDs in water, which is useful for biological applications. This spectrum also evidenced that the emission originates from the carbogenic core and that the tyrosine molecule is not in conjugation with the carbogenic core. Therefore, the electronic states of the carbogenic core are not perturbed after the addition of base. In order to understand the effect of base on CNDs in organic solvents, I re-recorded emission spectra of TCND-1 (10^−2^ mg/mL) in ethanol instead of acetone to avoid the Aldol condensation reaction between acetone molecules in the presence of base, which is shown in Figure 8b. This spectrum revealed that the said features for TCND-1 in acetone are also exhibited in ethanol. Three drops and eight drops of 0.4 M NaOH in ethanol was added to the same solution and a quenching of the emission intensity and a broadening of the band increment was observed at an excitation wavelength of 340 nm (Figure 8c).

The quenching of emission intensity reflects the ability of tyrosine-passivated CNDs for sensing ethoxide ions. Huang et al. reported that GQDs prepared from sucrose through microwave pyrolysis were applicable for sensing hypochlorous acid because a quenching of emission was observed [39]. Here, the quenching of the emission is because of the basic ethoxide ion (C_2_H_5_O^−^) being in contact with the excited state molecules, which affects the population of the excited state molecules and hence, results in a decrease of intensity and increase of the broadness of the band. This feature is also observed at other excitation wavelengths in the region between 280 and 460 nm (Figure S3 and Figure S4, See Supporting Information File 1).

**Figure 9:**
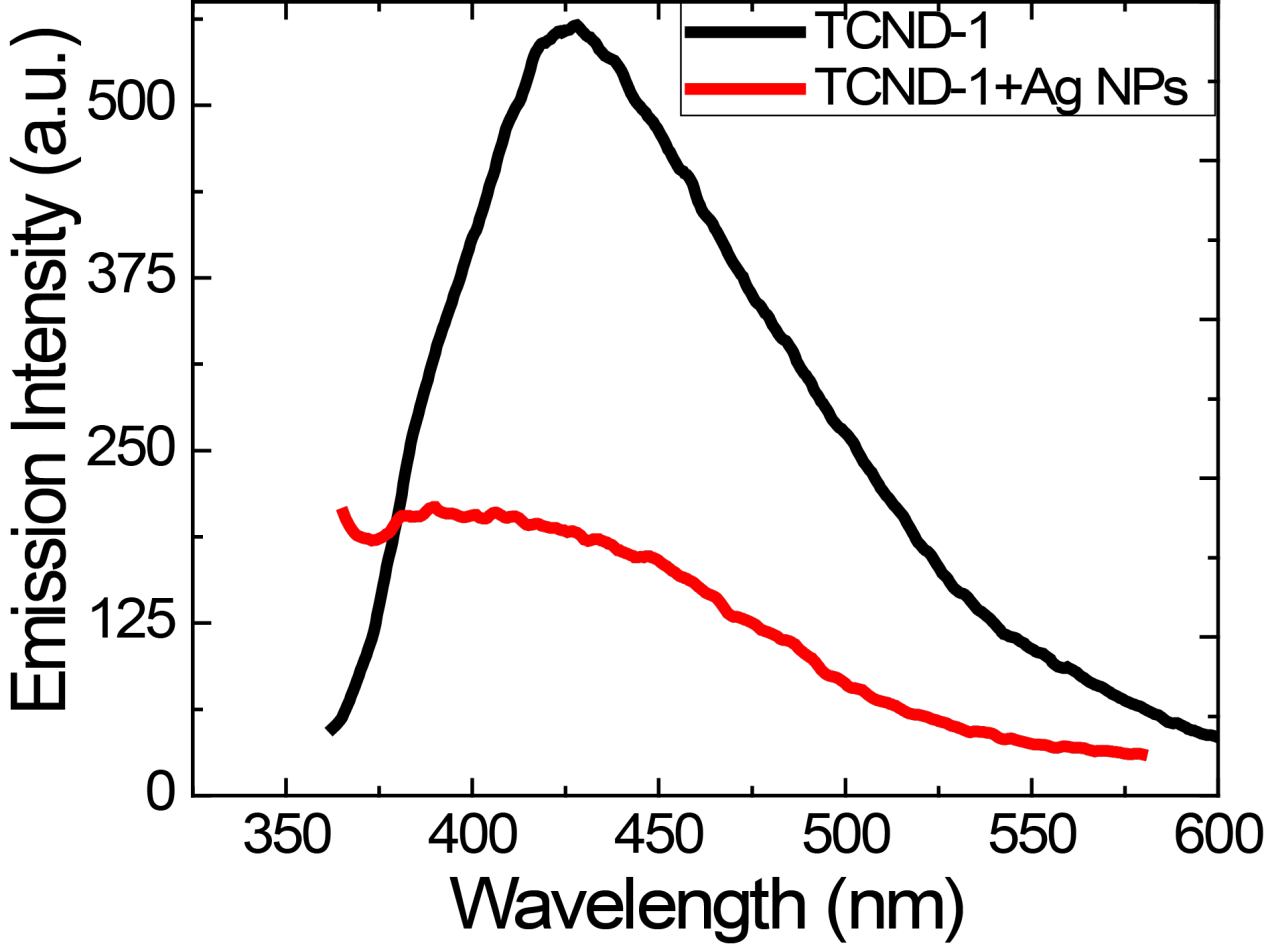
PL emission spectra of the aqueous TCND-1 solution and of the composite material solution, respectively, recorded at an excitation wavelength of 340 nm.

Sinha et al. reported the synthesis of Ag NPs by using sodium dodecyl sulfate (SDS) as both reducing and capping agent in water [40]. Freshly prepared Ag NPs solution (20 mL) with 60 mm SDS and 10^−3^ M Ag NO_3_ was mixed with 20 mL of aqueous TCND-1 solution (10 mg sample + few drops of 0.4 M NaOH) and subjected to stirring for 4 h. The absorption spectra of the resulting composite solution, the individual precursor solutions the colors of the solution before and after the reaction are shown in Figure 8d. The aqueous solution of TCND-1 exhibited two absorption bands centered at 294 nm and 340 nm (π–π* transition of conjugated -C=C-), respectively. The solution of the Ag NPs exhibited an absorption band centered at 490 nm with a tail extending to higher wavelengths [40] and the solution of the composite material exhibited an absorption band in the region of 330–490 nm centered at 420 nm which is related to surface plasmon resonance of spherical Ag NPs [41–42]. The blue shift of the absorption band of Ag NPs from 490 to 420 nm and the disappearance of the 340 nm band as well as the blue shift of absorption band of TCND-1 from 294 to 275 nm indicate the strong attractive interactions between the surface of the Ag NPs and TCND-1. Sinha et al. discussed the possible mechanism for the formation of Ag NPs when using SDS [40]. These attractive interactions are due to positively charged surface of the Ag NPs and the negatively charged surface of TCND-1 in aqueous solution. The emission spectra of the solutions of the composite material and of TCND-1 at an excitation wavelength of 340 nm are shown in Figure 9.

The composite material exhibited a reduced emission intensity compared with TCND-1 at an excitation wavelength of 340 nm. This indicates that the emission sites of TCND-1 are affected by the Ag NPs, which is also supported the disappearance of the absorption band at 340 nm in the absorption spectrum of the composite material (Figure 8d). It is known that the metallic surface induces a strong quenching of molecular fluorescence due to electromagnetic coupling between the metal and the fluorescent molecule [43]. Here, the reduction of the emission intensity indicates the ability of TCND-1 of sensing Ag NPs.

## Conclusion

In summary, I demonstrate a facile, simple approach to obtain fluorescent hydrophobic CNDs by using L-tyrosine and citric acid precursor molecules through a thermal oxidation process. During the carbonization process a selective transformation of tyrosine without disturbing its benzylic protons and the phenyl ring bearing the hydroxy group is observed. The synthesized CNDs have the ability of sensing ethoxide ions and Ag NPs. These tyrosine-passivated CNDs are soluble in organic solvents and in water under basic conditions. Due to this type of solubility the CNDs are applicable for both biological and commercial applications, which are under development.

## Experimental

### Materials

All chemicals were purchased from commercial sources and used without further purification. Citric acid was obtained from SD-Fine chemicals (SDFCL), L-tyrosine from Sisco Research laboratories (SRL. Pvt. Ltd), sodium hydroxide, and anthracene from MERCK chemicals. Solvents (spectroscopic grade) from Qualigens have been used for analysis. Deuterated dimethyl sulfoxide (DMSO-*d*_6_) was obtained from Sigma-Aldrich chemicals and used to record ^1^H NMR and ^13^C NMR spectra.

### Characterization techniques

Absorption spectra were recorded by using a Perkin-Elmer Lamda-35 spectrophotometer; Emission spectra were recorded by using a Perkin-Elmer LS-45 spectrophotometer at room temperature. IR spectra were recorded by using a BOMMEM-FTLA2000 spectrometer, ^1^H NMR and ^13^C NMR spectra were recorded by using a Bruker-Ultrashield 400 MHz spectrometer, transmisson electron microscope (TEM) images were recorded by using a JEOL-JEM2100 transmission electron microscope and diffraction patterns of the synthesized CNDs were recorded by using a Miniflex table top XRD, Rigaku/Japan.

## Supporting Information

File 1 Additional experimental data.
