# Supplementary material for: Synthesis of hydrophobic photoluminescent carbon nanodots by using L-tyrosine and citric acid through a thermal oxidation route
Source: Beilstein J Nanotechnol. 2014 Sep 11;5:1513–22. doi: 10.3762/bjnano.5.164 (PMC4168912; doi:10.3762/bjnano.5.164)
Supplement: File 1 — Additional experimental data. [file Beilstein_J_Nanotechnol-05-1513-s001.pdf]

# Supporting Information

for

# Synthesis of hydrophobic photoluminescent carbon nanodots by using L-tyrosine and citric acid through a thermal oxidation route

Venkatesh Gude*[§]

Address: Department of Chemistry, Assam University-Silchar, Assam, 788011-India.

Venkatesh Gude* - venkatesh.gude@gmail.com

* Corresponding author

[§] Phone: +919401048006

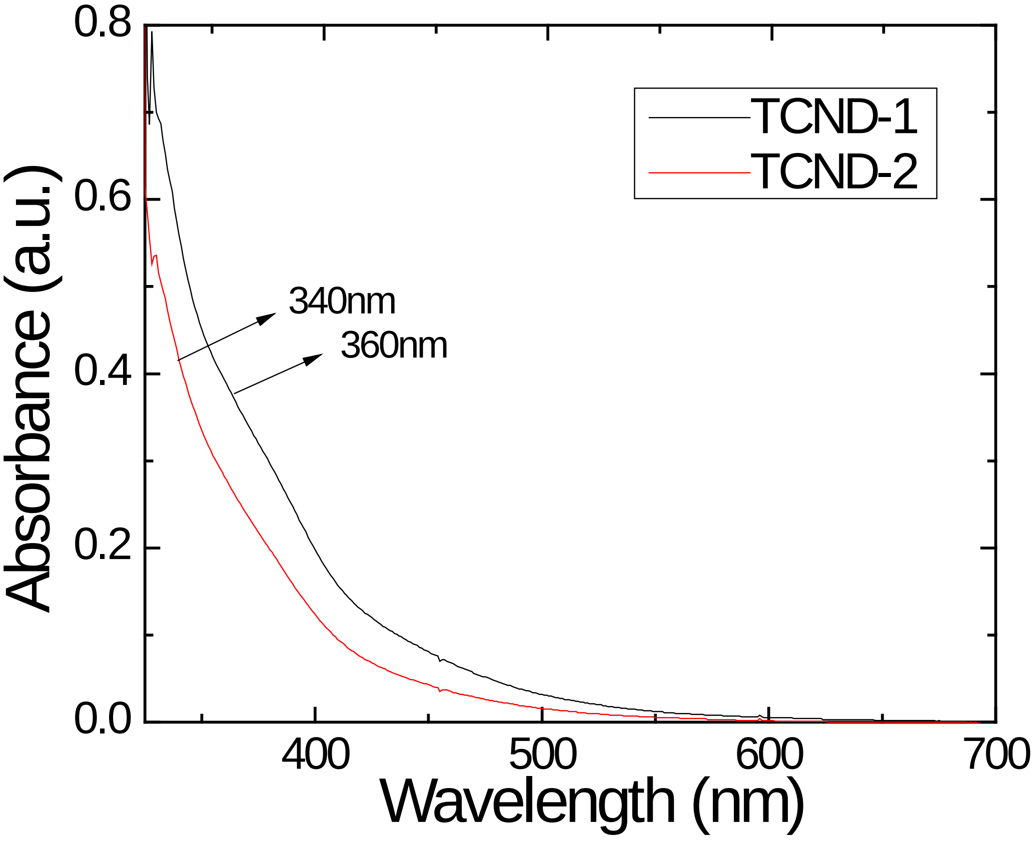


**Figure S1:** Absorption spectra of TCND-1 and TCND-2.

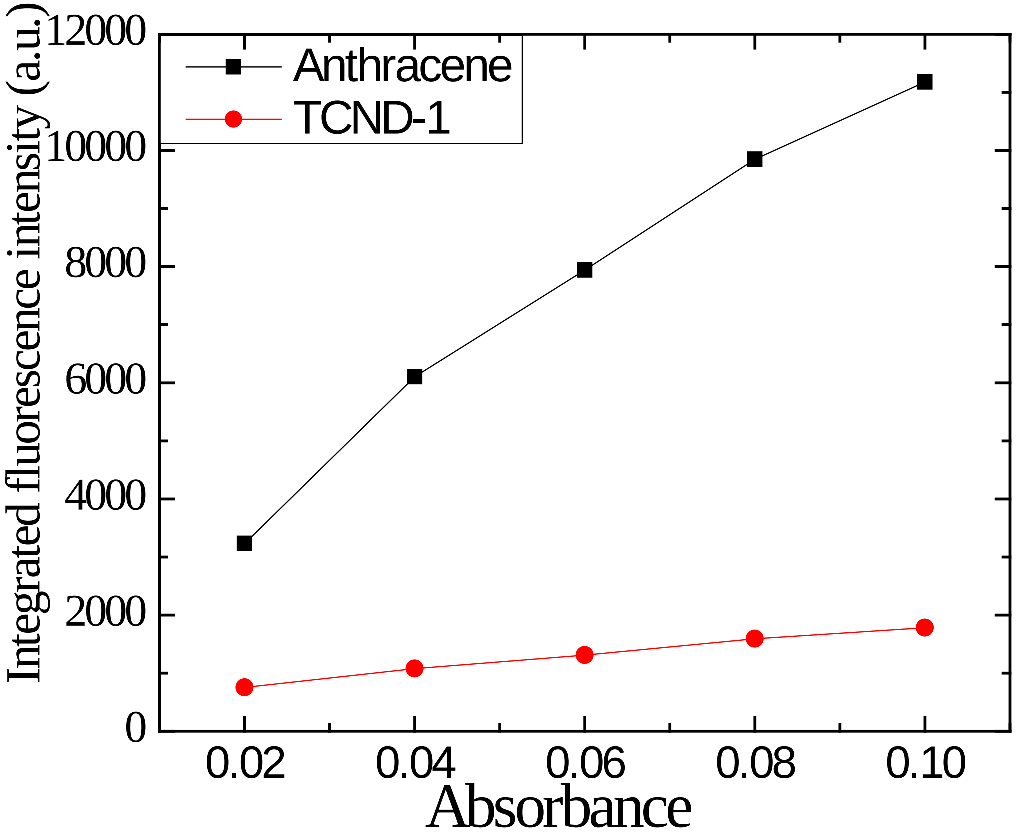


**Figure S2:** Plot for the measurement of the quantum yield of TCND-1 using anthracene as reference in ethanol at an excitation wavelength of 340 nm.

Formula used to measure the quantum yield (Φ) of TCND-1:

$$\Phi_{\text{TCND-1}} = \frac{\Phi_{\text{anthracene}} \times \text{gradient}_{\text{TCND-1}}}{\text{gradient}_{\text{anthracene}}}$$

$$= 0.27 \times \frac{\text{gradient}_{\text{TCND-1}}}{\text{gradient}_{\text{anthracene}}}$$

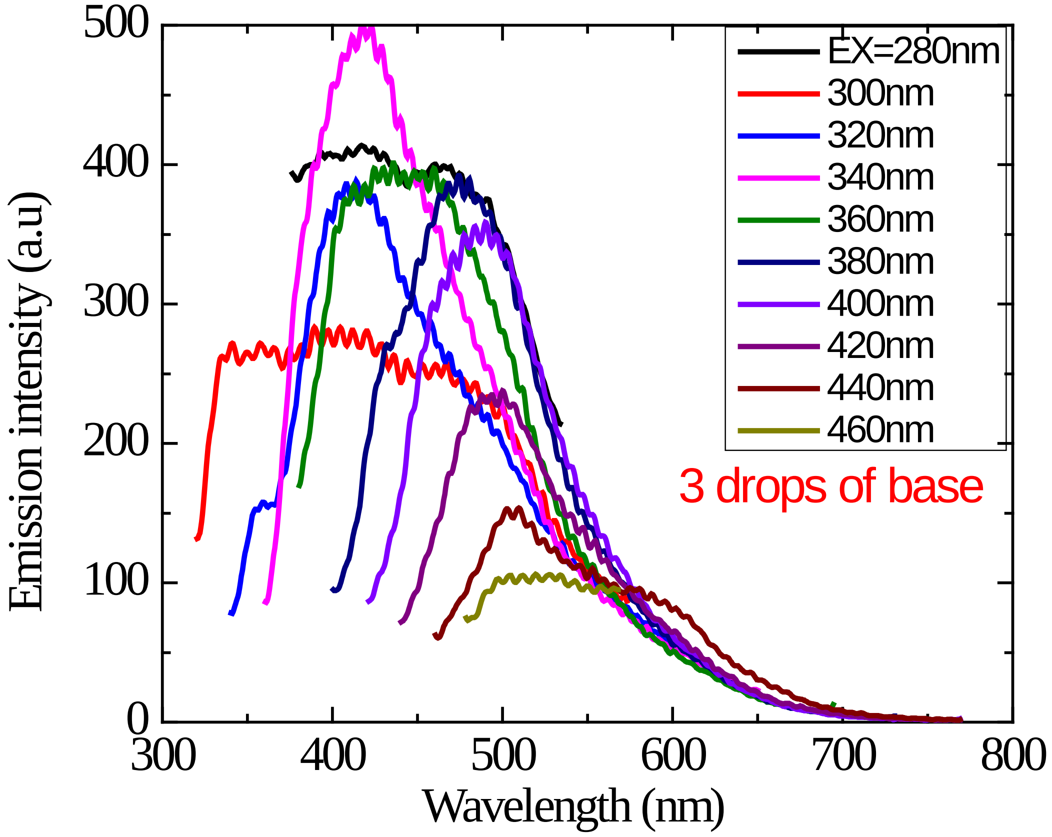


**Figure S3:** Recorded PL emission spectra of TCND-1 in ethanol after addition of 3 drops of base at different excitation wavelength.

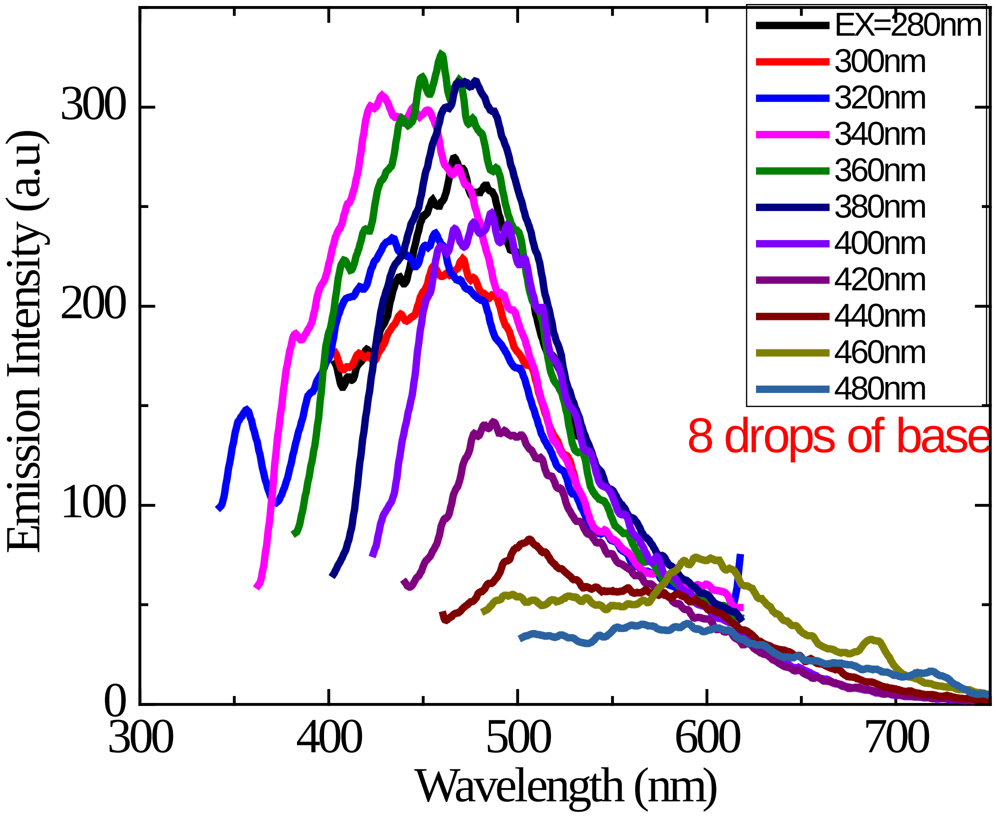


**Figure S4:** Recorded emission spectra of TCND-1 in ethanol after addition of 8 drops of base at different excitation wavelength.
